# CORRECTING GLUTATHIONE DEFICIENCY REVERSES MITOCHONDRIAL DYSFUNCTION AND ACCELERATED AGING IN PATIENTS WITH HIV

**DOI:** 10.1093/geroni/igz038.2025

**Published:** 2019-11-08

**Authors:** Rajagopal V Sekhar, Premranjan Kumar, James Suliburk, Charles G Minard, Chun Liu

**Affiliations:** Baylor College of Medicine, Houston, Texas, United States

## Abstract

Patients with HIV (PWH) have ‘accelerated’ aging based on early manifestation of geriatric comorbidities of declining physical-function, elevated inflammation, insulin-resistance, cognitive-impairment and abdominal-obesity, but contributing mechanisms are not well understood and interventions are lacking. We hypothesized that deficiency of the intracellular-antioxidant Glutathione results in impaired mitochondrial fuel-oxidation (MFO) and contributes to these defects, and that supplementing Glutathione precursors glycine and N-acetylcysteine (GlyNAC) could improve these defects. In an open-label trial, 8 PWH were studied before and after 12-weeks of GlyNAC supplementation (and 8-weeks after stopping GlyNAC), and compared to 8 matched, unsupplemented, uninfected controls. PWH had significantly impaired MFO, abnormal molecular regulation of MFO, muscle Glutathione deficiency, physical decline, cognitive-impairment, and higher oxidative-stress, inflammation, insulin-resistance and total body fat. GlyNAC supplementation significantly improved these defects, but benefits receded on stopping GlyNAC. These data suggest that GlyNAC supplementation could reverse ‘accelerated aging’ in PWH by improving defects linked to impaired MFO.

